# Computer-Aided Discrimination of Glaucoma Patients from Healthy Subjects Using the RETeval Portable Device

**DOI:** 10.3390/diagnostics14040349

**Published:** 2024-02-06

**Authors:** Marsida Bekollari, Maria Dettoraki, Valentina Stavrou, Dimitris Glotsos, Panagiotis Liaparinos

**Affiliations:** 1 Department of Biomedical Engineering, University of West Attica, Ag. Spyridonos, 12243 Athens, Greece; mbekolari@uniwa.gr (M.B.); dimglo@uniwa.gr (D.G.); 2 Department of Ophthalmology, “Elpis” General Hospital, 11522 Athens, Greece

**Keywords:** RETeval, glaucoma, machine learning

## Abstract

Glaucoma is a chronic, progressive eye disease affecting the optic nerve, which may cause visual damage and blindness. In this study, we present a machine-learning investigation to classify patients with glaucoma (case group) with respect to normal participants (control group). We examined 172 eyes at the Ophthalmology Clinic of the “Elpis” General Hospital of Athens between October 2022 and September 2023. In addition, we investigated the glaucoma classification in terms of the following: (a) eye selection and (b) gender. Our methodology was based on the features extracted via two diagnostic optical systems: (i) conventional optical coherence tomography (OCT) and (ii) a modern RETeval portable device. The machine-learning approach comprised three different classifiers: the Bayesian, the Probabilistic Neural Network (PNN), and Support Vectors Machines (SVMs). For all cases examined, classification accuracy was found to be significantly higher when using the RETeval device with respect to the OCT system, as follows: 14.7% for all participants, 13.4% and 29.3% for eye selection (right and left, respectively), and 25.6% and 22.6% for gender (male and female, respectively). The most efficient classifier was found to be the SVM compared to the PNN and Bayesian classifiers. In summary, all aforementioned comparisons demonstrate that the RETeval device has the advantage over the OCT system for the classification of glaucoma patients by using the machine-learning approach.

## 1. Introduction

Artificial intelligence (AI), based on machine-learning (ML) and deep learning (DL) techniques, has stimulated the scientific community in recent years in a variety of research domains, from basic investigation studies to industrial and clinical applications [1]. The methodology is developed using computational algorithms (a combination of computational power, neural networks and cloud storage), which process input data (via the extraction of various classes of features) with the purpose of recognizing structures and providing specific classification schemes within a decision-making framework with high accuracy [2,3,4,5].

In the direction of healthcare progress and improvement, AI computational studies have been used in different scientific investigations to provide a deeper understanding of the potential involvement of specific parameters in diagnosis, staging and prognosis prediction, and treatment, including clinical and technical challenges. Amongst the most important items in clinical indications are X-rays, chest [6,7] and breast abnormalities [8], skin malignant characteristics [9], brain diseases [10], cardiovascular risk factors [11], postoperative recovery aspects [12], etc. The necessity to identify pathological features in ocular diseases [13] has led to the spread of the implementation of AI in ophthalmology [14,15,16,17,18,19], where several diagnostic imaging techniques offer a comprehensive description of eye diseases based on morphological and functional feature datasets [20,21]. Nowadays, it is thought that there is a high association between visual function and human quality of life [22] since eye pathologies may degrade physical, emotional, and social activities [23]. In particular, AI has been used in ophthalmology for data analysis, segmentation, and automated diagnosis [15] and has played a critical role in outlining potential future pathways concerning eye wellness, providing breakthrough insightful information and outcome predictions of eye functionality. High-efficiency robust classification performance has been achieved in the detection of several ophthalmological diseases, like diabetic retinopathy, age-related macular degeneration, premature retinopathy, cataracts, anterior segment diseases, glaucoma, etc. [14,15,16,17,18,19]. Glaucoma is a common eye disease, which may become a chronic neuropathy and is globally recognized as a leading cause of visual impairment and blindness [24,25], leaving patients increasingly impaired in terms of daily activities. Our research, previous [26] and present, is focused on glaucoma patients since the early detection of abnormalities and, therefore, maintaining vision for as long as possible, which is a crucial parameter for this class of patients.

To combine AI capabilities in ophthalmology, several imaging modalities have been used, including the most commonly used methods and techniques, such as fundus photography, visual field (VF) testing, slit-lamp imaging, and optical coherence tomography (OCT) [14,15,16,17,18,19]. Lately, electroretinography (ERG) has been introduced as a significant functional and objective diagnostic tool that offers valuable information regarding the functionality of retinal ganglion cells and their axons [27]. In particular, it is the ophthalmologic test that provides quantitative measures of electrical activity in response to a light stimulus, which describes retinal neuron functionality. It has been shown that glaucoma patients with VF defects demonstrate pathologic photopic negative response (PhNR) values [28,29]. Traditional ERG testing necessitates controlled laboratory conditions and the cooperation of the patient within the framework of daily implementation in clinical settings [30]. The recent creation of a portable, handheld, and non-mydriatic full-field ERG system has the capability to address the mentioned limitations with future prospects to include such examinations in clinical routine since this portable device is fast, non-invasive, and patient-friendly [31,32].

In our previous study [26], we assessed the structural and functional changes in glaucoma patients and evaluated the correlation between the RETeval system’s parameters and the OCT parameters. We statistically analyzed and tested the parameters by using the SPSS.v28 software package. The study included patients with early glaucoma and healthy controls, and we further investigated the impact of age on the results. Statistical variances were observed in the age distribution among subgroups within the control group, particularly in relation to time–response parameters measured through the use of the RETeval system, although this discrepancy was not reflected in the OCT system. Furthermore, comprehensive comparisons between the case and control groups were conducted for both OCT and RETeval parameters, revealing some notable correlations. In the present article, we tried to extend our research by increasing the sample (both the glaucoma patients and the healthy participants) and by applying a machine-learning approach (different machine-learning algorithms) for the classification of glaucoma patients. Then, we further tried to demonstrate any possible classification differences based on eye selection (right and left) and gender (male and female) using both the OCT system and the portable RETeval device. Although there has been significant research on the use of AI in ophthalmology applications, the authors are not aware of AI investigations focusing on the RETeval device. 

## 2. Materials and Methods

### 2.1. Spectral-Domain OCT and RETeval Measurements

**Figure 1:**
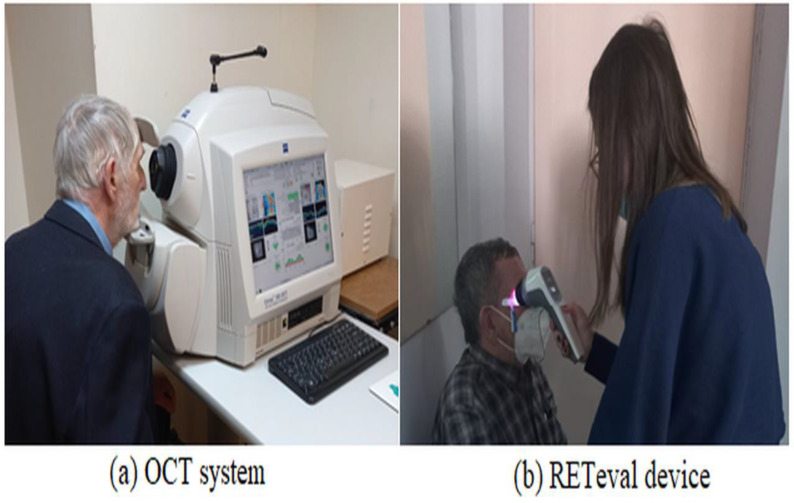
The figure shows the experimental setups used in our study. (**a**): the spectral-domain OCT (Cirrus HD-OCT 4000). (**b**): the portable RETeval recording device (LKC Technologies Inc.).

In this study, we used and compared two different experimental measurements obtained via (a) the OCT system and (b) the RETeval device for the detection of functional and structural changes in glaucomatous eyes. The OCT data were derived by using the Cirrus HD-OCT 4000 (Carl Zeiss Meditec Inc., Dublin, CA, USA), while the RETeval examination was carried out through the use of the portable device (LKC Technologies Inc., Gaithersburg, MD, USA), (Figure 1), as described previously [26]. 

**Figure 2:**
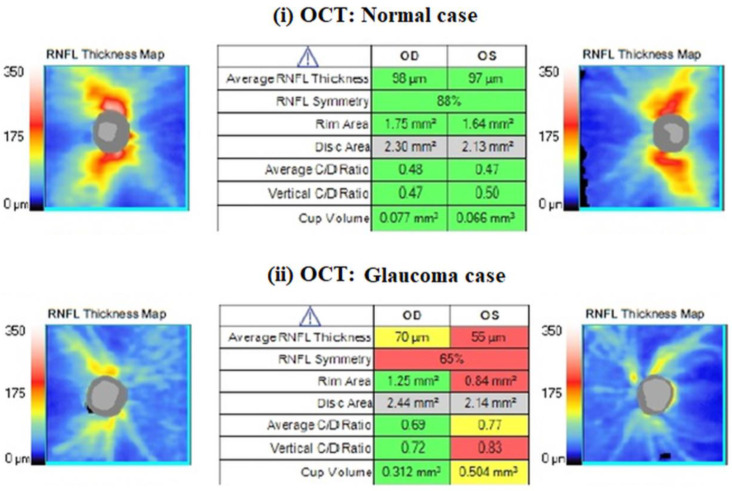
An example of a nerve fiber layer (RNFL) thickness map obtained with CIRRUS HD-OCT from ZEISS. The table format presents the key parameters in comparison to normative data, with shading in red, yellow, green, or white indicating their deviation from normal ranges. Red values are characterized as pathological, as we see in glaucoma patients.

**Figure 3:**
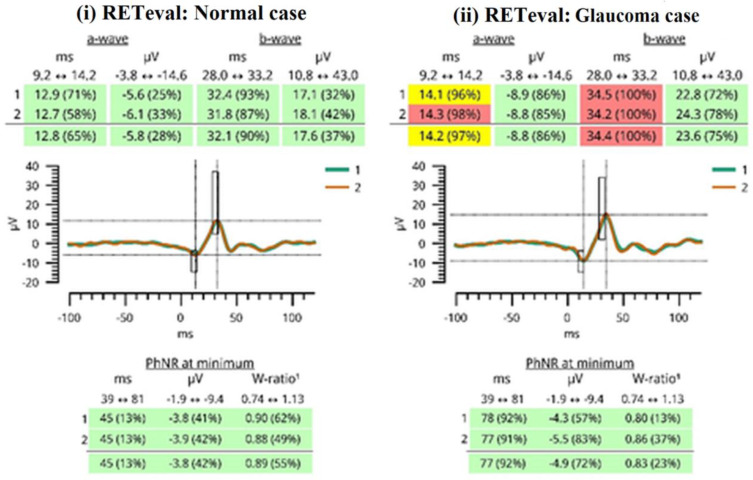
A report generated by the RETeval device is shown. Green color indicates that the results fall in the 95% of normal subjects. The yellow color indicates that the results fall in the next 2.5% of normal subjects. The red color indicates that the results fall outside of the “normal” 97.5% reference distribution percentile. The red results are considered pathological values, as seen in glaucoma patients.

One trained operator (V.S.) acquired the OCT images. We excluded the images with a signal strength of <6, media opacities, and those obtained during eye movements. For all participants, we used the Optic Disc Cube 200 × 200 protocol. The software exported and analyzed data on the thickness of the circumpapillary retinal nerve fiber layer (RNFL) on a circle with a diameter of 3.46 mm, centered on the optic disc center. The average circumpapillary RNFL thickness reflects the mean thickness across a 360-degree area surrounding the optic nerve head. Figure 2 shows the OCT images obtained by (i) a normal case and (ii) a patient with glaucoma in both eyes. On the other hand, photopic ERG measurements were obtained with the RETeval handheld device with the help of self-adhering skin sensor strip electrodes. The device provides a stimulus with a consistent retinal illuminance by adapting the luminance to account for variations in the pupillary area. The PhNR 3.4 Hz Td Long protocol involves delivering 200 flashes, with each set lasting approximately 60 s. The protocol includes a sequence of red flashes at 38 Td-s against a 380-Td blue background, with a stimulus frequency of 3.4 Hz. Subsequently, 400 sweeps were averaged for each recording. Prior to the measurement procedure, the participants underwent light adaptation in the clinical testing room for a minimum of 10 min. A list of parameters was evaluated as follows: (i) the a-wave amplitude (μV) and time response (ms), (ii) the b-wave amplitude (μV) and time response (ms), (iii) the minimum (Pmin) PhNR amplitude (μV) and implicit time (ms), and (iv) the W ratio. Analytical instructions concerning device instrumentation, protocol specifications, measurement acquirement, and parameter evaluation were provided in our previous article [26]. Figure 3 shows RETeval images obtained from (i) a normal case and (ii) a patient with glaucoma.

### 2.2. The Workflow of the Present Study

**Figure 4:**
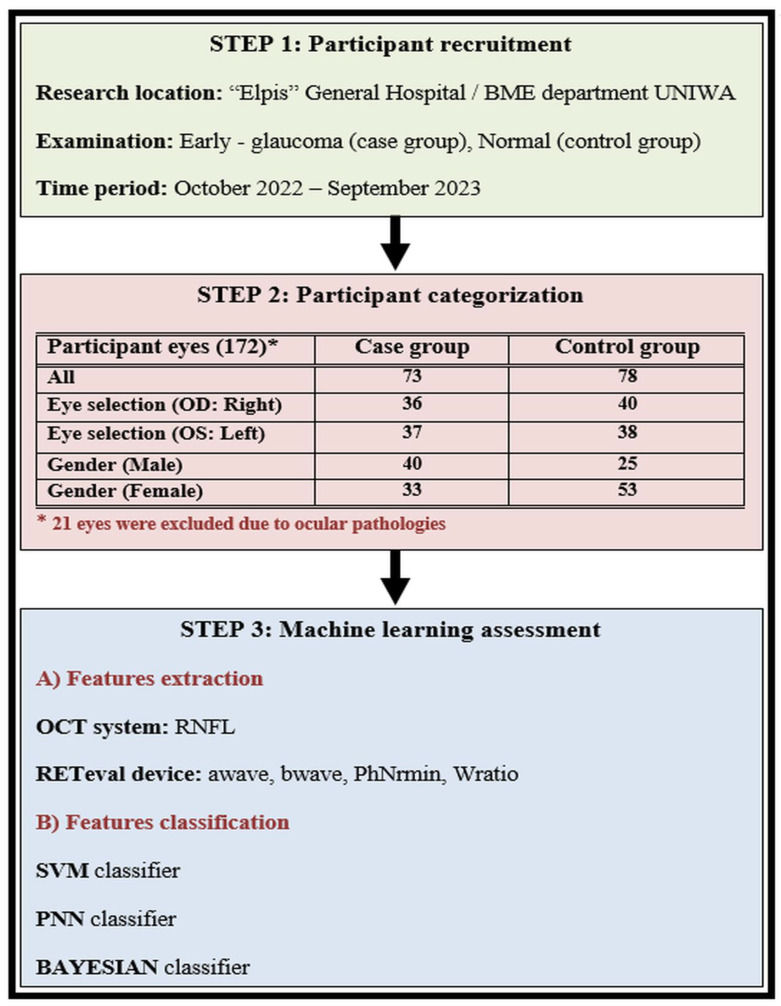
The workflow of our study is described in three steps: (1) participant recruitment, (2) participant categorization, and (3) the machine-learning approach.

The workflow of our investigation is illustrated in Figure 4 and includes three main stages: (i) participant recruitment, (ii) participant categorization, and (iii) the machine-learning approach.

Our investigation was carried out at the Ophthalmology Clinic of «Elpis» General Hospital through a memorandum of cooperation with the Biomedical Engineering Department of the University of West Attica in Greece. The time period of the patient examination was between October 2022 and September 2023, following all the required procedures according to the ethical approvals of both the University and General Hospital (Approval number 82608/19-09-2022), which adhered to the tenets of the Declaration of Helsinki. We recruited Caucasian individuals diagnosed with open-angle glaucoma who were currently undergoing ocular hypotensive therapy, forming what we refer to as the “case group”. Additionally, we included healthy Caucasian subjects with intraocular pressure (IOP) below 21 mmHg, normal optic nerve head appearance, and normal visual field (VF) test results or absence of other ocular pathology, constituting the “control group”. All participants underwent comprehensive ophthalmological examinations in both eyes, encompassing slit-lamp biomicroscopy, IOP measurements using Goldmann applanation tonometry, VF testing, as well as OCT and RETeval examinations. The identification of glaucomatous defects was conducted using the Zeiss Humphrey Field Analyzer 3, employing the Swedish Interactive Threshold Algorithm (SITA) standard test. Glaucomatous VF defects included a nasal step, generalized depression, hemifield defect, and inferior or superior paracentral or Bjerrum’s scotoma. All patients with glaucoma had primary open-angle glaucoma and were receiving treatment with topical hypotensive medication, either as a monotherapy or in combination with drugs such as prostaglandin analogs, alpha-2 agonists, beta-blockers, and carbonic anhydrase inhibitors. 

We conducted examinations on a total of 172 eyes, excluding 21 eyes for reasons such as a high degree of myopia, macular degeneration, and low OCT index outcomes. The rest of the 151 eyes were divided into two main groups: the case group with 73 eyes and the control group with 78 eyes. Thereafter, we tried to classify the two main groups by dividing and categorizing our samples into subgroups. The first subgroup was formed based on eye selection type (OD: right; OS: left). The right eye subgroup counted 36 (case) and 40 (control) participants, while the left eye subgroup counted 37 (case) and 38 (control) participants. The second subgroup was formed based on gender (male and female). The male subgroup counted 40 (case) and 25 (control) participants, while the female subgroup counted 33 (case) and 53 (control) participants. The main characteristics of the participants involved in our study are shown in Table 1. 

### 2.3. The Machine-Learning Approach

A machine-learning approach was developed to discriminate patients with glaucoma from the control cases. The machine-learning approach comprised three different classifiers: Bayesian, the Probabilistic Neural Network (PNN), and the Support Vectors Machines (SVMs). The Bayesian classifier is an optimal statistical classifier designed to minimize the probability error for data following a Gaussian distribution. Its discriminant function is expressed as shown in the following Equation [4]:

(1) $$d_{i}(x)=\ln(P(\omega_{i}))-\frac{1}{2}\ln(\left|C_{i}\right|)-\frac{1}{2}\left[{\left(x-m_{i}\right)}^{T}{C_{i}}^{-1}\left(x-m_{i}\right)\right]$$

In this context, *P(ω_i_)* denotes the probability of the occurrence of each class, *i*, *C_i_* stands for the covariance matrix, and *m_i_* represents the mean value of class *i*. The PNN classifier is a non-parametric four-layer feedforward neural network classifier. It calculates the probability density function (PDF) for each class by linearly combining the kernel PDF assessment for each training sample individually within a specific class. The discriminant function is defined as follows [2]:

(2) $$G_{j}(X)=\frac{1}{{\left(2\pi \right)}^{\left(\frac{n}{2}\right)}s^{n}N_{j}}\sum_{i=1}^{N_{j}}e^{\frac{-∥X-F_{j,i}∥}{2s^{2}}}$$

where *σ* denotes the spread of the Gaussian activation function, *N* denotes the number of pattern vectors, *d* denotes the dimensionality of the pattern vectors, and *x_ik_* denotes the *k*th pattern vector of class *i*. For our experiments, optimal performance was obtained for *σ* = 0.2. The SVM classifier aims to find the optimal mapping of the input space into a higher dimensional feature space, where the data can be linearly separable. It employs a non-linear transformation function (kernel), commonly using the Gaussian Radial Basis function. The discriminant function for binary classification problems is given as follows [3]:

(3) $$g(x)=\mathrm{sign}\left(\sum_{i=1}^{N}\alpha_{i}y_{i}K_{RBF}(x,x_{i})+b\right)$$

where *x_i_* represents the training data belonging to either class *y_i_* ∈ {+1,−1}, and *α_i_* and *b* are weight coefficients. For our experiments, the optimal performance for the RBF kernel was obtained for *σ* = 0.25. The selection of these classifiers was driven by their successful individual applications in various machine-learning applications in medicine, including the classification of OCT data [33,34,35]. The classifiers were trained using the exhaustive search and leave-one-out methods [4]. With the exhaustive search method, it is possible to assess the discrimination capacity for each different feature combination. The accuracy of each feature combination was computed using the leave-one-out method, according to which the classifier was designed with all cases but one. The left-one-out case was used to assess the classification accuracy of the classifier design. The process is repeated as many times as the total number of cases. Each individual classifier’s performance was optimized to maximize both the system’s sensitivity (detection of glaucoma cases) and specificity (detection of non-glaucoma cases). A custom MATLAB source code was employed in the development of all algorithms. 

## 3. Results

We initially tried to apply the three classifiers, SVM, PNN, and Bayesian, to test the classification accuracy between the control and the case group based on the RNFL thickness parameter in the OCT data. The classification accuracy was evaluated for the following: (i) all participants, (ii) eye selection (right and left), and (iii) gender (male and female), as provided in Table 2. Based on the numerical data, the following particular outcomes can be subtracted. The classification accuracy was found to be (a) exactly similar (81.1%) for the three classifiers in the sample of all participants, (b) the SVM and Bayesian classifiers were more efficient for the classification of the eye selection and the gender, (c) to be higher in terms of examining the right eye compared to the left eye. The highest difference was estimated to be 14% for SVM classifier, and (d) to be higher examining the female sample compared to male one. The highest difference was assessed to be 8.8% again for SVM classifier.

We further examined the classification accuracy based on the RETeval device data by testing different feature combinations of the parameters listed in the Section 2. 

**Figure 5:**
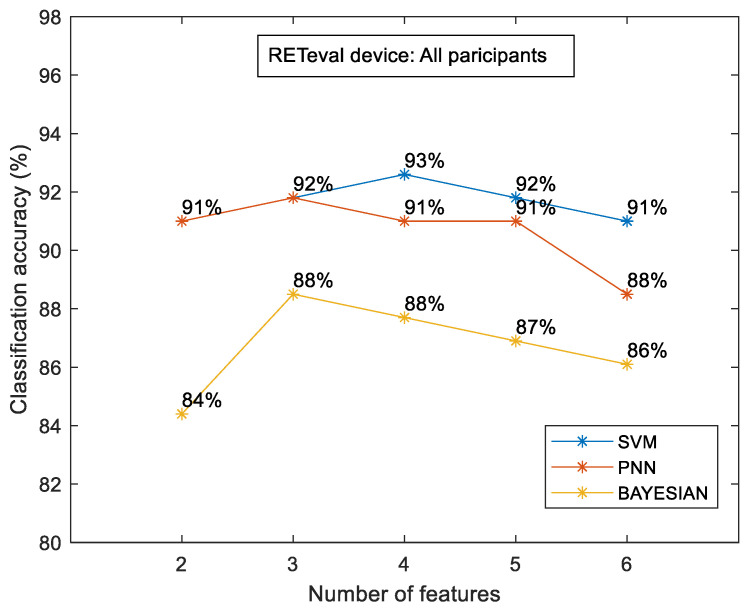
RETeval classification accuracy between the case and the control group for all participants. The results are provided for SVM, PNN, and Bayesian classifiers.

**Figure 6:**
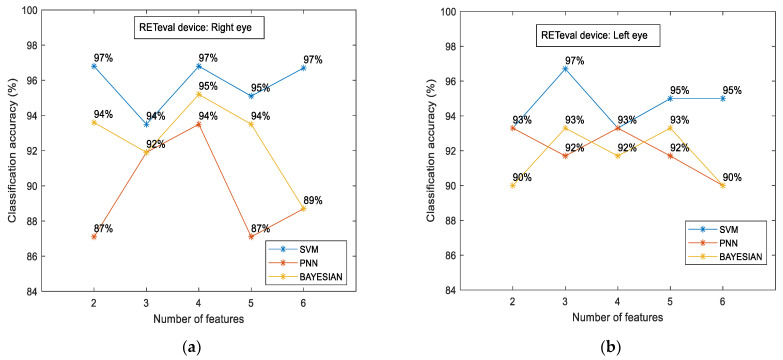
RETeval classification accuracy between the case and the control groups in terms of eye selection: right (**a**) and left (**b**). The results are provided for SVM, PNN, and Bayesian classifiers.

Figure 5 shows the classification accuracy between the control and the case groups for the sample of all participants for each classifier separately. The most efficient classifier was the SVM classifier compared to the PNN and Bayesian classifiers. However, all classifiers showed higher accuracy in relation to the OCT numerical data. The highest classification accuracy was calculated to be approximately 93% (for the combination of four (4) features), which was 14.7% higher than the OCT-evaluated accuracy (81.1%). Figure 6 shows the classification accuracy between the control and the case group in terms of eye selection: (i) right and (ii) left. For both eyes, no significant differences were observed. In particular, the SVM classifier was the most efficient classifier, with approximately 97% accuracy in both cases. Furthermore, the RETeval data provided higher classification accuracy with respect to the OCT numerical data, which was specifically 13.4% for the right eye and 29.3% for the left eye. 

**Figure 7:**
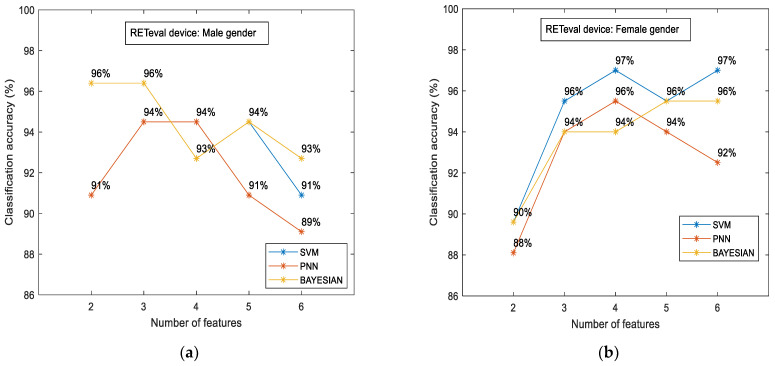
RETeval classification accuracy between the case and the control group in terms of gender: male (**a**) and female (**b**). The results are provided for SVM, PNN, and Bayesian classifiers.

To examine if there were variations in the measured characteristics between the case and control groups in terms of gender, we additionally examined the classification accuracy for the male and female subgroups separately. The results are illustrated in Figure 7. 

The main findings of such comparisons are given as follows: (i) the classification accuracy was slightly higher in the female subgroup (97%) in contrast to the male subgroup (96%), and (ii) the aforementioned accuracy was achieved with different classifiers. SVM was the most efficient classifier for the female subgroup, as in all cases mentioned above. On the other hand, the highest accuracy for the male subgroup was evaluated through the Bayesian classifier, and, for the first time, (iii) the RETval results showed higher accuracy in relation to the OCT numerical data. This difference was estimated to be 25.6% for the male gender (from 76.4% up to 96%) and 22.6% for females (from 79.1% up to 97%).

## 4. Discussion

Glaucoma treatment is considered to be one of the main domains in ophthalmology since this particular ocular disease degrades the quality of daily life and may lead to gradual visual damage and total blindness in patients [25]. The pathogenesis of glaucoma is not fully understood [36] and it generally has a slow and asymptomatic progression until its advanced stages [26]. Therefore, it is crucial to proceed to an accurate diagnosis as early as possible in order to stand the progression of glaucoma and maintain vision as long as possible. There are several already existing methods, tests, and systems for such purposes, such as VF testing and the OCT system. However, recently new diagnostic tools have been developed to examine the optic nerve and monitor the functionality of retinal ganglion cells, such as the RETeval device [26], which became commercially available in 2014. Since then, various investigations have shown that the RhNR, obtained via ERG, seems promising in terms of detecting glaucoma [37], even for the assessment of different stages of optic neuropathy [32] or in eyes with diagnostic dilemmas [27]. The RETeval device has also been used for other optic nerve disorders [38] (such as optic neuritis, ischemic optic neuropathy, traumatic optic neuropathy, and dominant optic atrophy), diabetic retinopathy [39,40,41], and retinal vein occlusions [42,43] as well as for chronic brain disorders, such as schizophrenia [44,45]. Studies have also been conducted on healthy pediatric populations in order to establish normative electroretinogram values [46,47]. 

We tried to examine the role of portable RETeval parameters in glaucoma diagnosis and investigate their relationship to glaucoma patients (case group) compared with those of healthy individuals (control group). Until now, there have been no reference data concerning the use of RETeval analysis data through AI algorithms that may classify patients with glaucoma with higher accuracy than other conventional examinations (e.g., the OCT examination). The main finding of our study, for all cases examined, was the remarkably higher classification accuracy observed in processing the RETeval data with respect to the data obtained from the OCT system. The calculated differences were quite significant: 14.7% for all participants, 13.4% and 29.3% for eye selection (right and left, respectively), and 25.6% and 22.6% for the gender case (male and female, respectively). This evidence is very important for the application of the machine-learning approach to RETeval numerical data in glaucoma patients. We believe that these classification scores are due to the possibility of extracting a plethora of parameters using the RETeval device. For example, the classification accuracy examining the sample of all participants was achieved for the combination of four (4) features. The peaking phenomenon [48] of feature selection was also present in our study. That means that the highest accuracy was not achieved by using the highest number of feature combinations. Therefore, further research is required to achieve a better understanding of which parameters are most suitably combined in order to increase the classification accuracy. In any case, the classification accuracy was even better for the RETeval device by combining the lowest number of extracting parameters (i.e., the combination of two (2) parameters). Apart from accuracy, in order to enhance our results, we provide additional data (sensitivity, specificity, and the area under the ROC curve—AUC), which are summarized in Table 3. 

While a direct comparison with previous studies is difficult due to variations in [49,50,51,52] (a) experimental settings and equipment for data acquisition (e.g., the type of optical detection modality used, the number of patients, the analysis of structural or functional parameters, the stage of glaucoma, the different treatment criteria, etc.) and (b) different methodologies for data processing and analysis (e.g., the usage of supervised or unsupervised algorithms, the choice of machine or deep learning approaches, the type of classifiers, the number of extracted features, etc.), our results align with other reported results based on supervised ML approaches in the existing literature [53,54], as shown in Table 3.

**Table 3:** A summary of studies using machine-learning (ML) classifiers for glaucomatous and healthy subjects. The results are provided in terms of sensitivity, specificity, and the area under the ROC curve (AUC).

Authors (Year)	Number of Subjects or Eyes	ML Classifiers	Derived Data	Results
Our study, (2024)	73 glaucoma eyes; 78 healthy eyes	Support Vector Machine (SVM)	RETeval data	Sensitivity: 89.8% Specificity: 95.2% AUC: 0.911
Our study, (2024)	73 glaucoma eyes; 78 healthy eyes	Probabilistic Neural Network (PNN)	RETeval data	Sensitivity: 83.1% Specificity: 96.8% AUC: 0.864
Our study, (2024)	73 glaucoma eyes; 78 healthy eyes	Bayesian	RETeval data	Sensitivity: 88.1% Specificity: 87.3% AUC: 0.857
Singh et al. [55], (2021)	70 glaucoma eyes; 70 healthy eyes	K-Nearest Neighbor (KNN)	OCT data	Sensitivity: 100% Specificity: 87.5% AUC: 0.970
Lu et al. [56], (2018)	40 glaucoma eyes; 64 healthy eyes	Logistic regression (LR)	Biomechanical data	Sensitivity: 98.9% (at 80% specificity) Sensitivity: 97.7% (at 95% specificity) AUC: 0.990
Salam et al. [57], (2016)	26 glaucoma subjects; 74 healthy subjects	Support Vector Machine (SVM)	OCT data	Sensitivity: 100% Specificity: 87%
Barella et al. [58], (2013)	57 glaucoma eyes; 46 healthy eyes	Tree-based ensembled model	SD-OCT data	Sensitivity: 64.9% (at 80% specificity) Sensitivity: 49.1% (at 90% specificity) AUC: 0.877
Silva et al. [59], (2013)	62 glaucoma subjects; 48 healthy subjects	Tree-based ensembled model	SD-OCT and SAP data	Sensitivity: 95.2% (at 80% specificity) Sensitivity: 82.2% (at 90% specificity) AUC: 0.946
Garcia-Morate et al. [60], (2009)	136 glaucoma eyes; 117 healthy eyes	Support Vector Machine (SVM)	HRT2 data	Sensitivity: 85.3% (at 75% specificity) Sensitivity: 79.4% (at 90% specificity) AUC: 0.905
Nayak et al. [61], (2009)	37 glaucoma subjects; 24 healthy subjects	Neural network classifier	OCT data	Sensitivity: 100% Specificity: 80%
Townsend et al. [62], (2008)	140 glaucoma eyes; 60 healthy eyes	Support Vector Machine (SVM)	HRT3 data	Sensitivity: 85.0% (at 85.7% specificity) Sensitivity: 64.8% (at 95% specificity) AUC: 0.904
Burgansky-Eliash et al. [63], (2005)	47 glaucoma eyes; 42 healthy eyes	Support Vector Machine (SVM)	OCT data	Sensitivity: 97.9% (at 80% specificity) Sensitivity: 92.5% (at 92.5% specificity) AUC: 0.981
Zangwill et al. [64], (2004)	95 glaucoma eyes; 135 healthy eyes	Support Vector Machine (SVM)	HRT data	Sensitivity: 97% (at 75% specificity) Sensitivity: 85% (at 90% specificity) AUC: 0.964
Goldbaum et al. [65], (2002)	156 glaucoma eyes; 189 healthy eyes	Support Vector Machine (SVM)	SAP data	Sensitivity: 53% (at 100% specificity) Sensitivity: 71% (at 90% specificity) AUC: 0.903

Another important finding of our study was the identical classification accuracy in terms of eye selection (approximately 97%) with the RETeval device, while there was a discrepancy in the OCT data (85.5% for the right eye and 75% for the left, respectively). This result (a) verifies the statistical analysis (through *T*-test and Mann and Whitney U tests) of our previous study [26] between the right and left eye for both groups (case and control) where no statistical differences were found, and (b) enhances the future glaucoma research by increasing the sample via the collection of data from both eyes. In addition, a significant contribution of our work is related to the RETeval study on a particular population (the Caucasian population) and a specific eye disease (glaucomatous optic neuropathy). It has been proposed that there are racial factors that affect the estimation of glaucomatous damage of the optic nerve (like differences in optic disc appearance, RNFL thickness, and central corneal thickness) and, therefore, no reliable comparisons can be made between different racial groups [66,67,68,69] or even between groups that are characterized with different glaucoma severity categories [32,70]. This might be an advantage regarding the reliability of our results since our research is more concrete to a particular group of population and focuses on specific glaucoma characteristics; however, at the same time, it could be characterized by a limited overall assessment of glaucoma disease. Further limitations of the sample have been provided in our previous article [26] in regard to the following: (i) the correlation of the optic disc between the two systems (RETeval and OCT), (ii) the follow-up of patients, (iii) the implementation of standard automated perimetry (SAP) data in the overall comparison assessment, and the (iv) further examination of schemes (age, pupil diameter, etc.). However, a limitation of our study is the lack of focus on a specific stage of glaucoma since our patients were characterized by varying severity in terms of glaucomatous neuropathy. Nevertheless, all glaucoma patients had primary open-angle glaucoma, and their glaucomatous defects were verified via both VF testing and RNFL damage. Further limitations of the sample have been provided in our previous article [26] in regard to the following: (i) the correlation of the optic disc between the two systems (RETeval and OCT), (ii) the follow-up of the patients, (iii) the implementation of standard automated perimetry (SAP) data in the overall comparison assessment, and (iv) further examination of schemes (age, pupil diameter, etc.). 

The authors realize that there are many obstacles to overcome in developing AI for glaucoma diagnosis in clinical practice. For example, the criteria for glaucoma diagnosis should be standardized since the spectrum of glaucoma is wide and complex, and the patterns of dealing with the disease are slightly different for each glaucoma expert [51]. The treatment criteria for glaucoma also differ. According to a recent report [51], for more accurate performance, it is pivotal to compare and standardize glaucoma diagnostic data at as many centers as possible and train AI models based on this verified dataset. Furthermore, the evaluation of glaucoma progression requires the analysis of time-series and multimodal data of patients, including IOP measurements, fundus photography, OCT, and VF sensitivity. Therefore, various strategies are required to develop AI for diagnosing, monitoring, and treating glaucoma, and future prospective validation studies are crucial to evaluating the accuracy and reliability of glaucoma screening AI models in real-world settings [71].

## 5. Conclusions

A machine-learning approach, through the use of different classification algorithms, has been used to examine the capabilities of the RETeval device in the indication of glaucoma patients. Further examination was carried out to assess additional characteristic effects, such as eye selection and gender. The numerical data obtained from the RETeval device showed considerably higher classification accuracy compared to the OCT system. Further examination of the most appropriate combination of features is required; however, we believe that our study introduces a significant contribution to the machine-learning methodology involving ophthalmological RETeval data, and it could serve as a valuable supplementary tool for the objective examination and analysis of patients diagnosed with glaucoma. 

## Figures and Tables

**Table 1:** The characteristics of the participants involved in this study. All data are expressed as mean value ± standard deviation.

	Mean Value ± Standard Deviation					
Variables	Case Group (*n* = 73)	Male (*n* = 40)	Female (*n* = 33)	Control Group (*n* = 78)	Male (*n* = 25)	Female (*n* = 53)
Age (years)	67.5 ± 11.2	65.8 ± 13.3	69.0 ± 7.9	52.7 ± 11.5	51.9 ± 10.3	54.4 ± 13.2
RNFL thickness (μm)	75.2 ± 12.9	74.7 ± 13.7	75.8 ± 12.1	92.0 ± 8.5	90.2 ± 7.3	92.9 ± 9.0
a-wave implicit time (ms)	12.9 ± 1.0	12.8 ± 11.0	12.9 ± 0.8	12.0 ± 0.8	11.8 ± 0.6	12.1 ± 0.9
a-wave amplitude (μV)	−7.5 ± 2.3	−7.1 ± 2.6	−7.9 ± 1.9	−7.7 ± 2.5	−5.6 ± 1.0	−8.7 ± 2.3
b-wave implicit time (ms)	30.6 ± 2.1	30.4 ± 2.4	30.8 ± 1.7	28.7 ± 1.4	28.0 ± 1.5	29.1 ± 1.3
b-wave amplitude (μV)	20.8 ± 6.1	19.7 ± 6.4	22.2 ± 5.5	23.2 ± 6.5	18.2 ± 3.3	25.5 ± 6.2
PhNrmin (ms)	60.7 ± 9.2	60.1 ± 8.2	61.4 ± 10.4	57.4 ± 10.0	53.5 ± 12.7	59.8 ± 7.9
PhNrmin (μV)	−4.4 ± 1.4	−4.2 ± 1.3	−4.7 ± 1.4	−5.0 ± 2.3	−4.5 ± 1.1	−5.1 ± 2.6
Wratio	0.9 ± 0.1	0.9 ± 0.1	0.9 ± 0.1	0.9 ± 0.1	0.9 ± 0.1	0.9 ± 0.1
IOP (mm Hg)	15.5 ± 2.8	15.9 ± 3.2	15.1 ± 2.4	15.7 ± 2.0	16.8 ± 1.5	15.3 ± 2.1

**Table 2:** OCT classification accuracy between the case and the control group. Results are provided for SVM, PNN, and Bayesian classifiers and concern the following: (i) all participants, (ii) right eye selection, (iii) left eye selection, (iv) male gender, and (v) female gender.

OCT Classification Accuracy between Control and Case Group			
Participants	SVM Classifier	PNN Classifier	Bayesian Classifier
All	81.1%	81.1%	81.1%
Eye (OD: Right)	85.5%	74.2%	83.9%
Eye (OS: Left)	75.0%	69.1%	75.0%
Gender (Male)	72.7%	69.1%	76.4%
Gender (Female)	79.1%	62.7%	79.1%

## Data Availability

Data analysis is contained within the article. Anonymized data presented in this study are only available upon reasonable request due to the ethical approval statement.
